# Investigation of Exhaled Breath Samples from Patients with Alzheimer’s Disease Using Gas Chromatography-Mass Spectrometry and an Exhaled Breath Sensor System

**DOI:** 10.3390/s17081783

**Published:** 2017-08-03

**Authors:** Hui-Chong Lau, Joon-Boo Yu, Ho-Won Lee, Jeung-Soo Huh, Jeong-Ok Lim

**Affiliations:** 1 Biomedical Research Institute, Department of Biomedical Science, Kyungpook National University, 50 Samduk, 2 Ga, Jung-Gu, Daegu 700-721, Korea; huichong.lau@gmail.com; 2 Department of Materials Science and Metallurgical Engineering, Kyungpook National University, Sankyuk-dong, Puk-Gu, Daegu 41566, Korea; jyacht@naver.com; 3 Department of Neurology, Kyungpook National University, 50 Samduk, 2 Ga, Jung-Gu, Daegu 700-721, Korea; neuromd@knu.ac.kr

**Keywords:** exhaled breath, alzheimer’s disease, gas sensor, exhaled breath sensor system, gas chromatography–mass spectrometry

## Abstract

Exhaled breath is a body secretion, and the sampling process of this is simple and cost effective. It can be non-invasively collected for diagnostic procedures. Variations in the chemical composition of exhaled breath resulting from gaseous exchange in the extensive capillary network of the body are proposed to be associated with pathophysiological changes. In light of the foreseeable potential of exhaled breath as a diagnostic specimen, we used gas chromatography and mass spectrometry (GC-MS) to study the chemical compounds present in exhaled breath samples from patients with Alzheimer’s disease (AD), Parkinson’s disease (PD), and from healthy individuals as a control group. In addition, we also designed and developed a chemical-based exhaled breath sensor system to examine the distribution pattern in the patient and control groups. The results of our study showed that several chemical compounds, such as 1-phenantherol and ethyl 3-cyano-2,3-bis (2,5,-dimethyl-3-thienyl)-acrylate, had a higher percentage area in the AD group than in the PD and control groups. These results may indicate an association of these chemical components in exhaled breath with the progression of disease. In addition, in-house fabricated exhaled breath sensor systems, containing several types of gas sensors, showed significant differences in terms of the normalized response of the sensitivity characteristics between the patient and control groups. A subsequent clustering analysis was able to distinguish between the AD patients, PD patients, and healthy individuals using principal component analysis, Sammon’s mapping, and a combination of both methods, in particular when using the exhaled breath sensor array system A consisting of eight sensors. With this in mind, the exhaled breath sensor system could provide alternative option for diagnosis and be applied as a useful, effective tool for the screening and diagnosis of AD in the near future.

## 1. Introduction

Human exhaled breath has been proposed as a potential specimen following efforts to search for reliable non-invasive samples for diagnostic purposes. Exhaled breath odors have been used since ancient Greek times as part of the physical examination to diagnose a disease [1,2]. Eventually, studies have found that the distinctive odors from exhaled breath can provide clues in the diagnosis of disease [2,3]. In the modern era, with the emergence of science and technology, the analysis of exhaled breath has raised clinical interest. In addition, exhaled breath sampling is rather easy to handle, cost effective, and can be obtained more easily than other commonly used specimens [3,4,5].

The composition of exhaled breath is directly associated with the circulation of gases from the whole body to the capillary networks of the alveolus prior to exhalation [6,7]. The chemical composition of exhaled breath samples varies between subjects due to genetic factors, infection, medication, and individual lifestyle [3,4,6,8]. It has also been proposed that the chemical compounds in exhaled breath could originate from the inhalation of contaminants from the environment [4,8], and be produced from the blood, peripheral tissue, and lungs as a result of biochemical reactions in the body [4,6,7]. As a disease progresses, its pathological processes are found to have a significant impact on the production of gases, either by producing new types of gases or by the metabolic consumption of the gases [7,8].

To date, exhaled breath samples have been widely studied in various types of diseases, such as lung-related diseases, infectious diseases, diabetes, breast cancer, prostate cancer, inflammatory disease, and others [9,10,11,12,13,14]. Several studies have reported a correlation between the gases of exhaled breath and metabolic pathways [2,3,7,8]. Hence, there is great potential for the use of exhaled breath as a non-invasive specimen to study neurodegenerative diseases, especially Alzheimer’s disease (AD). The analysis of exhaled breath samples from patients with AD could be an alternative option, since there is no direct and definitive single diagnostic method. Previously, a study described the use of nanomaterial gas sensors to distinguish patients with AD from healthy individuals with an accuracy of 85% [15]. Thus, the further analysis of exhaled breath samples from patients with AD may also provide useful insights into the development of this disease.

In this study, we analyzed exhaled breath samples from patients with AD, Parkinson’s disease (PD), and from healthy persons using gas chromatography–mass spectrometry (GC-MS) and in-house fabricated exhaled breath sensor systems. We studied the chemical compounds in the exhaled samples to determine those distinctive to the AD group. We included PD patients in the study because PD is the second most common neurodegenerative disease after AD. It has a different mechanism, signs, and symptoms from those seen in patients with AD. As a result, patients with PD can be used as a group distinguishable from those in the AD group. With the limited data available on exhaled breath analysis for patients with AD, studies on the distribution pattern and chemical composition of exhaled breath from this group are of great interest and value. Exhaled breath compounds could provide fundamental information on AD progression. Additionally, an exhaled breath system could represent an alternative screening method for AD.

## 2. Materials and Methods

### 2.1. Collection of Exhaled Breath Samples from Participants

The method of collecting human exhaled breath was reviewed and approved by the institutional review board (IRB) of the Kyungpook National University Hospital (IRB code KNUH_12-1002). Signed consent forms were obtained from all of the participants recruited in this study. The personal information of participants was protected by giving each exhaled breath sample a specific code number before being used in the study.

**Figure 1:**
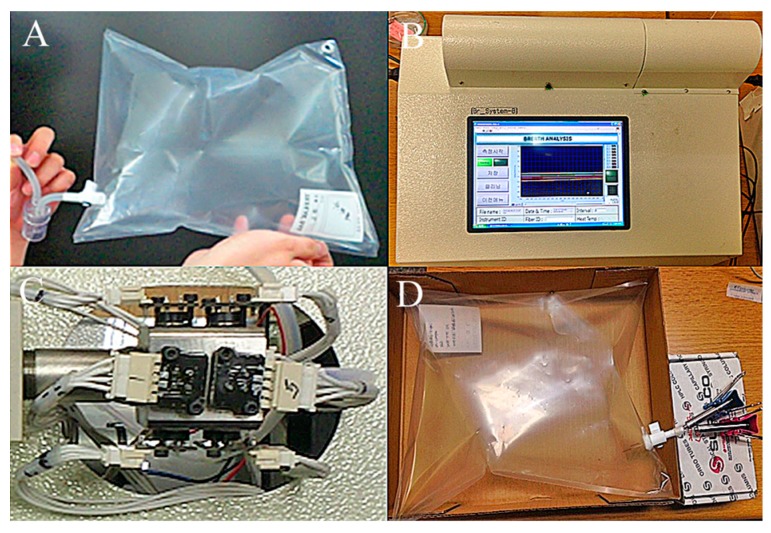
Collection and analysis of exhaled breath. (**A**) Collection of exhaled breath into a 3 L Tedlar bag using a mouth-piece; (**B**) Exhaled breath sensor system for clustering exhaled breath samples; (**C**) Gas sensors placed on the metal-oxide semiconductor; (**D**) Pre-concentration of chemical compounds in the exhaled breath samples using solid phase microextraction (SPME) fiber.

Sixty human exhaled breath samples, including 40 from the AD and PD patient groups and 20 from healthy individuals, were collected from Kyungpook National University Hospital. Participants were requested to fast for at least 4 h before providing 3 L of their exhaled breath into a Tedlar bag through a mouth-piece filter (Figure 1A). All AD and PD group participants were regular follow-up patients from the hospital, whereas the control group members included individuals with good general health and no history of other neurological, psychiatric, or major medical diagnosis that could contribute significantly to cognitive impairment or dementia. All participants were also clinically examined using neuropsychological and clinical tests prior to their inclusion in the study. The results of the neuropsychological tests, such as the mini-mental state examination (MMSE) and the clinical dementia rating scale sum of boxes (CDR-SOB), conducted on the patient and control groups were distinctively different. The patients with AD had an MMSE score of 17.95 ± 4.86, whereas the scores for patients with PD and healthy individuals were 22.85 ± 4.65 and 28.55 ± 1.05, respectively. The patients with AD had the highest CDR-SOB score of 6.1 ± 2.6 compared to those with PD and healthy individuals, for which the scores were 2.24 ± 2.67 and 0.25 ± 0.30, respectively. The Hoehn–Yahr (H-Y) score was only applicable for PD patients, at 2.31 ± 0.56. The details of participant data and clinical scores are listed in Table 1.

### 2.2. Design of Solid Phase Microextraction (SPME)

Generally, the concentration of exhaled breath is within the range of parts per billion or trillion. As a result, pre-concentration is necessary in order to maximize the concentration of chemical substances. Coated fiber, similar to the one used in solid phase microextraction (SPME), is used to pre-concentrate the chemical compounds from exhaled breath prior to analysis using GC-MS and the exhaled breath sensor systems. The SPME fiber used in this study was obtained from Sigma, St. Louis, MO, US. It was coated with 65 μM polydemethysiloxane/divinylbenzene (PDMS/DVB), and contained a hydrophilic and hydrophobic layer to allow the absorption and desorption, respectively, of chemical compounds.

### 2.3. Characterization of Chemical Compounds Present in Exhaled Breath Using GC-MS

Randomly selected exhaled breath samples were analyzed using GC-MS within 24 h at room temperature of receiving the samples. Despite the short term storage at room temperature, there is a chance of composition change of exhaled breath. Nine such samples from the AD and control groups and five from the PD group were used to identify the specific chemical compounds present in each sample. The analysis was performed on an Agilent, 5975C Inert XL MSD (quadrupole MS) coupled with a 6890A gas chromatograph (Agilent, Waldbron, Germany) with a split/splitless injector. The temperature of the split/splitless injector was 250 °C. Helium was used as a carrier gas with the linear velocity set at 36.6 cm/s. The MS analysis was carried out in full scan mode, with a scan range between 1.6 and 0.50 amu. The column length was 30 cm and the diameter 0.25 mm × 0.25 μm. The mass range was 29–550 *m*/*z*. The oven temperature program was as follows: initial temperature at 60 °C for 1 min, then ramped at 7 °C/min to 300 °C for 20 min. The transfer line and ionization source were heated to 250 °C, and the quad temperature was 190 °C with ionization energy of 70 eV.

The SPME fiber was first injected into the exhaled breath to absorb the chemical compounds for 30 min. The SPME fiber with the extracted analytes was then inserted into the split/splitless injector of the GC-MS system and heated to 250 °C. The chemical compounds were thermally desorbed in the splitless mode for 5 min and then analyzed using GC-MS. All chemical compounds were identified and confirmed by using retention time and mass spectral library match. Each identified compound was measured as percentage area to represent the proportion in relation to the total area of the peaks detected. The degree of similarity between the spectrum of a compound and the MS spectrum of known compounds in the library was indicated as percentage of quality. Only peaks with a match quality of more than 80% were used in the analysis. The presence of chemical compounds from exhaled breath in terms of the percentage area of the GC-MS spectra were compared between the AD, PD, and control groups in order to identify the chemical compounds that could be used to correlate with disease progression. An occurrence of a chemical compound with less than 50% in each group will not be included in the analysis in order to obtain more reliable and valid data for comparison. The average of the percentage area for each respective compound in each group was calculated in order to determine the potential chemical compound in the exhaled breath from patients with AD.

### 2.4. Clustering Analysis of Exhaled Breath Samples Using an Exhaled Breath Sensor System

The exhaled breath sensor system consists of a chamber to locate the fibers, a data acquisition system, a processor of several nanostructure metal oxide gas sensors, and a program such as principal component analysis (PCA) or Sammon’s mapping to analyze the data from the sensor system (Figure 1B). We fabricated two types of exhaled breath analyzing systems, sensor array system A and sensor array system D, each with a combination of different types of sensors. In system D, four types of gas sensor, including the TGS 2600, TGS 2602, TGS 2611, and TGS 2620 (Figaro, Osaka, Japan), were used. In contrast, system A consisted of eight types gas sensor, including the TGS 2600, TGS 2602, TGS 2610 (Figaro, Osaka, Japan), MICS 5135, MICS 5132, MICS 2610, and MICS 2610 (e2V Technologies, Chelmsford, Essex, UK), of which the MICS 5135 was used twice in this system (Figaro, Osaka, Japan). These sensors are capable of detecting odorous and volatile organic gases. The metal oxide gas sensors were placed on SnO_2_-based thin film sensors deposited with Au, Cr, or Wo, developed by INFM-CNR, Italy, and the Physics Institute, Lithuania (Figure 1C).

To study the distribution clustering of the AD, PD, and control groups, the SPME fiber was first directly exposed to the exhaled breath samples collected in the Tedlar bag at an ambient temperature for 30 min (Figure 1D). The analytes extracted and concentrated by the fiber were then injected into the splitless mode of both of the exhaled breath systems at 250 °C for 5 min to desorb the analytes. The analysis was performed by determining the change of the sensitivity characteristics of the metal oxide sensors using a pattern analysis program. The normalized responses of the sensitivity characteristics were used for the subsequent clustering analysis. Twenty samples each from the AD, PD, and control groups were analyzed using three different clustering methods: PCA, Sammon’s mapping, and a combination of both methods. These methods were able to provide distinctive patterns to distinguish human odors based on the eigenvector calculation and Euclidean distance measurement. The use of PCA and Sammon’s method were previously described and studied for odor patterning in agricultural applications [16]. We used these methods to analyze the distribution cluster of exhaled breath for the control and patient groups.

### 2.5. Statistical Tests

Data obtained from GC-MS and the exhaled breath sensor systems were analyzed using GraphPad Prism Software version 5.00 (GraphPad Software, San Diego, CA, USA). Results are expressed as mean ± standard deviation. Differences between the AD, PD, and control groups were determined using one-way analysis of variance (ANOVA). Values were considered significantly different when the calculated *p*-value was ≤0.05.

## 3. Results and Discussion

Based on the retention time and spectral search analysis, we found four types of chemical compounds that were consistently present in all of the exhaled breath samples of the patient and control groups (Table 2). We used percentage areas of compounds for the analysis in this study. This is because at this stage, our study aimed to screen for those exhaled breath compounds which are responsible for AD. As a result, we did not carry out additional experiments to calibrate the compounds. Based on the compounds, we found the exhaled breath gas sensor for an optimum detection purpose. The analysis on chemical compounds of an independent exhaled breath sample from an AD patient, a PD patient, and a healthy individual, respectively, is shown in Table 1. The analysis of the percentage area of the peaks found that phenol showed no difference between the groups. A previous study [15] reported the presence of elevated alkane and methylated alkanes groups in AD and PD groups. Additionally, benzene, which was found elevated in the AD and PD groups in the previous report, is highly associated with DNA damage. In our study, alkane and benzene groups were also found with higher percentage areas than other compounds, but not with a statistically significant difference between the studied groups. In contrast, we observed a statistically significant difference from Ethyl 3-cyano-2,3-bis (2,5-dimethyl-3-thienyl)-acrylate, triphenyl phosphate, and 1-phenanthrenol.

Acetamide had a higher percentage area of the spectrum in patients with AD than those in the PD and control groups. In particular, lower levels of acetamide were observed in the PD group than in the control group. Although the difference was small, it was significant. One study also reported the use of acetamide as a novel acetyl cholinesterase inhibitor, which could potentially be used to treat AD [17]. Perhaps, levels of exhaled acetamide could be associated with the progression of this disease.

In addition to those four compounds found consistently in all the exhaled breath samples, there were an additional four types of chemical compounds present in more than 50% of the samples in each group. These included 1,2-benzenedicarboxylic acid diethyl ester, triphenyl phosphate, 1-phenantherol, and ethyl 3-cyano-2,3-bis (2,5,-dimethyl-3-thienyl)-acrylate (Table 2). These compounds were identified and confirmed using retention time and spectral library match. The percentage areas of the spectrum for 1-phenantherol and ethyl 3-cyano-2,3-bis (2,5,-dimethyl-3-thienyl)-acrylate were significantly higher in patients with AD than those in the PD and control groups. As their occurrence was low in the control group, the percentage areas of the spectrum for ethyl 3-cyano-2,3-bis (2,5-dimethyl-3-thienyl)-acrylate and 1-phenantherol were not determined. It was previously reported that the presence of 1-phenanthrenol is possibly attributed to endocrine disruption or accumulation of acetylcholinesterase at the synapse, and that it further disrupts the function of the nervous system and impairs behavior in a fish model [18,19]. Hence, this could suggest an association of high levels of 1-phenanthrenol and the occurrence of AD. In addition, no ethyl 3-cyano-2,3-bis (2,5-dimethyl-3-thienyl)-acrylate was found in the PD group. Although there have been no specific reports on the role of ethyl 3-cyano-2,3-bis (2,5-dimethyl-3-thienyl)-acrylate in the development of neurodegenerative diseases, it could be an indicator to distinguish between the AD and PD groups. Further investigation is necessary to provide better insight into the correlation between exhaled ethyl 3-cyano-2,3-bis (2,5-dimethyl-3-thienyl)-acrylate and the occurrence of AD.

Moreover, 1,2-benzenedicarboxylic acid diethyl ester had a higher percentage area of the spectrum in patients with AD than those in the PD and control groups, whereas patients with PD had lower levels than those in the control group, although the differences were not statistically significant. A previous study showed that the use of this compound induced exitotoxicity and neuronal cell death [20]. Furthermore, it may also contribute to the impairment of memory as a consequence of declining choline acetyltransferase function [20]. In contrast, one study also demonstrated the potential use of 1,2-benzenedicarboxylic acid dinonyl ester in reducing Aβ-induced neurotoxicity [21]. Hence, further studies of this compound in exhaled breath could be useful.

Additionally, triphenyl phosphate had a higher percentage area of the spectrum in healthy individuals than in patients with AD. Despite the difference being insignificant, these results may suggest that disease progression reduces the production of triphenyl phosphate in exhaled breath. Triphenyl phosphate has been proposed as being useful in combating the diseases involving the irreversible inhibition of acetylcholinesterase [22]. A previous report also described the potential use of the isomers of triphenyl phosphate, such as dialkyl phenyl phosphate, as inhibitors of cholinesterase to slow down the progression of AD [23].

**Figure 2:**
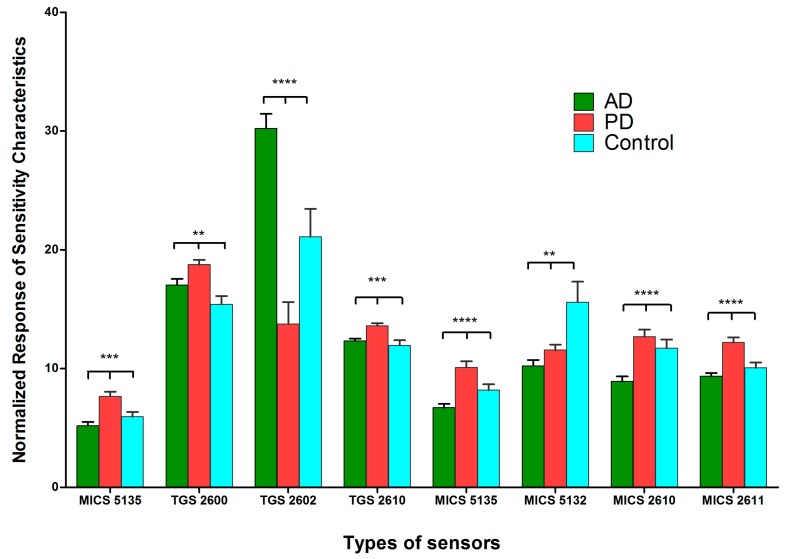
Normalized responses of the sensitivity characteristics for different types of gas sensors (System A). Response sensitivity of exhaled breath samples from the patients with Alzheimer’s disease (AD), Parkinson’s disease (PD), and from healthy individuals as a control group to gas sensors. The data are represented as mean ± standard deviation. The *p*-value was calculated using one-way analysis of variance (ANOVA). Differences were considered significant when the calculated ** *p* ≤ 0.01, *** *p* ≤ 0.001, and **** *p* ≤ 0.0001.

**Figure 3:**
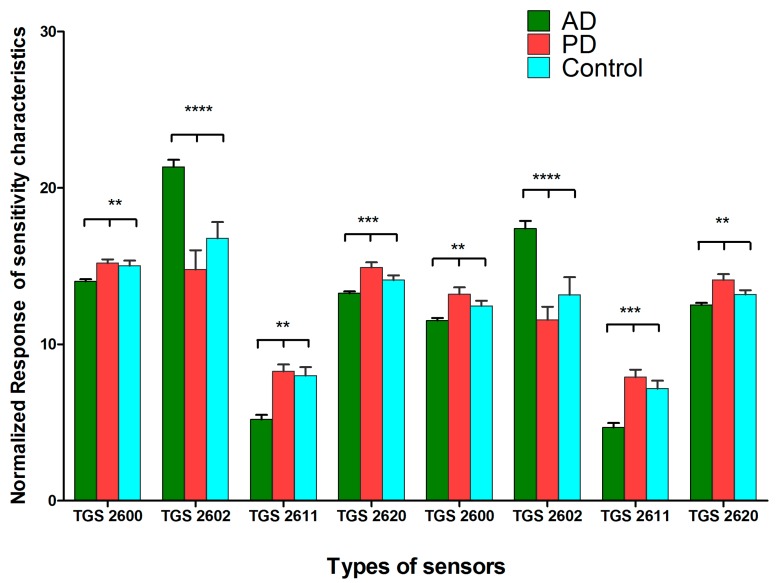
Normalized responses of the sensitivity characteristics for different types of gas sensors (System D). Response sensitivity of exhaled breath samples from patient and control groups to gas sensors. The data are represented as mean ± standard deviation. The *p*-value was calculated using one-way analysis of variance (ANOVA). Differences were considered significant when the calculated ** *p* ≤ 0.01, *** *p* ≤ 0.001, and **** *p* ≤ 0.0001.

Furthermore, to distinguish the exhaled breath samples between the patient and control groups, we carried out an exhaled breath clustering analysis using the exhaled breath sensor systems. The normalized responses of the sensitivity characteristics for the gas sensors in system A (Figure 2) and D (Figure 3) were significantly different between the AD, PD, and control groups. System A, with TGS 2602, MICS 5135, MICS 2610, and MICS 2611, had distinctive sensitivity characteristics, whereas TGS 2602 from system D was found to have significantly different sensitivity characteristics in the AD, PD, and control groups. The TGS 2602 sensor from both systems had distinctive differences between the patient and control groups. The normalized response of the sensitivity characteristics of TGS 2602 in the AD group was higher than that in the PD and control groups. The TGS 2602 sensor is designed to detect volatile organic compounds and odorous gases. By comparing the results to the GC-MS analysis, polyaromatic hydrocarbons, such as 1-phenantherol, were also found to have high percentage areas of the GC-MS spectrum in the AD group. This may imply a correlation between the findings from the exhaled breath systems and the GC-MS analysis. Additionally, TGS 2600, MICS 2610, MICS 2611, and MICS 5135 had higher levels in patients with PD than those in the AD and control groups. Some of these gas sensors, such as MICS 5135 and MICS 2610, had high normalized sensitivities in the detection of silicone vapor. Since silicone is produced by silicon and oxygen, this may suggest a relationship between high siloxane in the exhaled breath and the high normalized response sensitivity of the gas sensors used with the PD group.

**Figure 4:**
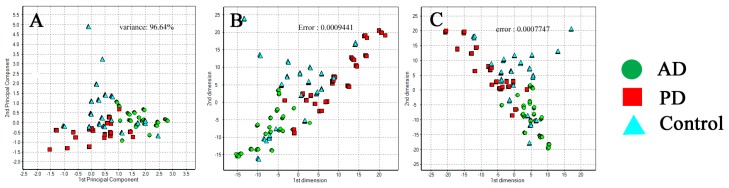
Distribution clusters for exhaled breath samples of Alzheimer’s disease (AD), Parkinson’s disease (PD), and control groups using exhaled breath system A. (**A**) Principal component analysis (PCA) clustering method for AD, PD, and control groups; (**B**) Sammon’s mapping for AD, PD, and control groups; (**C**) Combination of PCA and Sammon’s mapping for patient and control groups.

**Figure 5:**
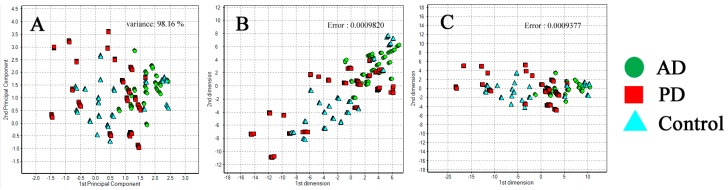
Distribution of clusters for exhaled breath samples from patients with Alzheimer’s disease (AD), Parkinson’s disease (PD), and from healthy individuals as a control group using system D. (**A**) Principle component analysis (PCA) pattern analysis of AD, PD, and control groups; (**B**) Sammon’s mapping of AD, PD, and control groups; (**C**) Combination of PCA and Sammon’s mapping for healthy individuals, AD patients, and PD patients.

Further clustering analysis of the exhaled samples showed that the AD, PD, and control groups were patterned into three clusters in system A (Figure 4) and system D (Figure 5). Our results demonstrated that the control group could be distinguished from the AD and PD patient groups based on the PCA and Sammon’s mapping methods with a confidence limit of 95%. The PCA method showed a percentage of variance of 96.64% and 98.16% in system A and D, respectively. The Sammon’s mapping method had error rates of 9.441 × 10^−4^ and 9.820 × 10^−4^ in system A and D, respectively. The combination of both methods had error rates of 7.747 × 10^−4^ and 9.377 × 10^−4^ in system A and D, respectively. Using more types of sensors, the exhaled breath sensor system A showed better distribution patterns in the AD, PD, and control groups. The distinctive composition variations in the exhaled breath samples of the patient and control groups are most likely attributable to the discrete clusters. Some of the healthy individuals clustered with the patient groups were those who had or previously had smoking habits. In addition, personal eating habits and environmental factors may also cause a healthy individual to be clustered with those from the patient groups.

Despite the variances in the chemical compositions observed from the exhaled breath samples, the source and origin of most of the chemical compounds in the exhaled breath remains unknown. Moreover, the specific pathways and biochemical reactions contributing to the differences in chemical composition are also undetermined. Perhaps, the development of neurodegenerative diseases contributes to increased levels of these chemical compounds in exhaled breath via the alteration of chemical reactions in the body. Moreover, effects of medication and life style could also vary the composition of exhaled breath, especially for patients with AD and PD, since long-term drug therapy might change the chemical compositions of exhaled breath.

Oxidative stress is also one of the vital key processes influencing the chemical composition of exhaled breath [24,25]. Oxidative damage to the cells, protein, nucleic acids, sugars, and even lipids has been previously reported in patients with AD [26,27]. An elevation of specific biomolecule markers associated with oxidative stress has also been intensively studied in patients with AD. It has been reported dyshomeostasis contributes to elevated zinc, copper, and iron in patients with AD [28,29,30]. Subsequently, the dysregulation and imbalance of metal homeostasis in AD could change the metabolite by-products in the body [28]. These changes may contribute to the variation of chemical compounds in exhaled breath samples.

We also correlated the chemical compounds from the exhaled breath with potential metabolic pathways based on the chemical structures of the compounds. Chemical compounds such as 1-phenantherol and ethyl 3-cyano-2,3-bis (2,5-dimethyl-3-thienyl)-acrylate belong to the group of hydrocarbons. Hydrocarbons are mostly produced by lipid peroxidation, and are in the form of secondary metabolites or intermediate products in the body [31]. One study has shown that silicon can be secreted in bile, and subsequently it affects the lipid metabolism of the body. As a result, lipid metabolism disorders may contribute to the increase of exhaled siloxane in patients with PD.

**Figure 6:**
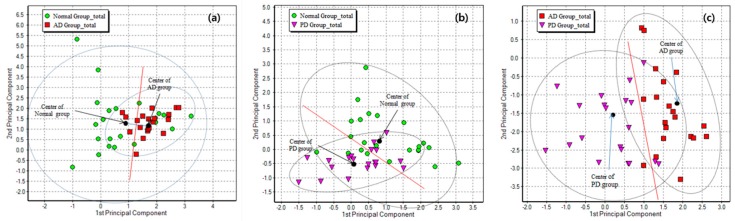
Distribution of clusters by Principle component analysis (PCA) pattern analysis for exhaled breath samples from patients with Alzheimer’s disease (AD), Parkinson’s disease (PD), and from healthy individuals as a control group using system A. (**a**) AD patients and healthy individuals; (**b**) PD patients and healthy indivisulas; (**c**) AD patients and PD patients.

The results from the GC-MS and exhaled breath sensor system analyses of exhaled breath samples suggest that the chemical compounds present in the patient groups are significantly different from those of healthy individuals. Based on these findings, patients in the AD group could be distinguished from those in the PD and control groups based on the clustering analysis using the exhaled breath sensor systems. The distribution pattern is associated with the variations in chemical compositions of the exhaled samples, which result from pathophysiological changes in the body. Furthermore, it is also possible that the accumulated peroxidative damage and pollutants previously exposed to the body are released gradually in the exhaled breath when the defense system of a body becomes weak [32]. The distribution of clusters by Principle component analysis (PCA) pattern analysis for exhaled breath samples from patients with Alzheimer’s disease (AD), Parkinson’s disease (PD), and from healthy individuals as a control group using sensor array system A is expressed in Figure 6. Because there is no definitive evidence of the mechanisms and origin of the chemical components in exhaled breath, further study of such factors in patients with AD is necessary. Additionally, a longitudinal study should be carried out to verify the consistency and reliability of composition variation in the exhaled breath of patients with AD. Despite this, the findings from our exhaled breath study using GC-MS and sensor systems suggest that the exhaled breath system could potentially be used as a diagnostic tool for AD in the near future. The current development of chemical sensors is restricted to the external sensing of the compound of interest. In the near future, a chemical sensor ought to be explored as an ingestible sensor to improve health services and ensure a better quality of life [33].

## 4. Conclusions

Our results represent a potentially new screening approach for exhaled breath samples using the exhaled breath sensor system. We developed a gas sensor array system by utilizing principal component analysis, Sammon’s mapping, and a combination of both methods to distinguish the exhaled specimens collected from patient and control groups. Using the variations in composition of the exhaled breath from the patient and control groups, the breath sensor system could distinguish patients with AD from those with PD and healthy individuals into distinctive clusters. The normalized responses of the sensitivity characteristics for System A were significantly different between the AD, PD, and control groups. This conclusively suggests the potential use of the exhaled breath sensor system as a screening tool for AD in the near future.

## Figures and Tables

**Table 1:** Demographic and clinical data of the participants in the study.

Characteristic	AD N (20)	PD N (20)	Control N (20)	*p*-Value
**Age, (Y)**	74.9 ± 7.53	72.3 ± 7.55	67.6 ± 7.03	-
**Male sex-no (%)**	30	50	25	-
**Smoker (%)**	10	5	10	-
**Ex-smoker (%)**	5	20	10	-
**MMSE**	17.95 ± 4.86	22.85 ± 4.65	28.55 ± 1.05	≤0.05
**CDR-SOB**	6.1 ± 2.6	2.24 ± 2.67	0.25 ± 0.30	≤0.05
**H-Y**	nd	2.31 ± 0.56	nd	-

Values denoted as mean ± standard deviation. Scores on the mini-mental state examination (MMSE) range from 0 (severe impairment) to 30 (no impairment). A score higher than 27 was considered normal. The clinical dementia rating scale sum of boxes (CDR-SOB) scores range from 0 (cognitive normality) to 18 (maximal cognitive impairment). Hoehn–Yahr (H-Y) scores from stage 1 (mild) to stage 5 (maximal disabling). The *p*-value for variables was computed using one-way analysis of variance (ANOVA). *p ≤* 0.05; ns = not significant; nd = not determined; AD, Alzheimer’s disease; PD, Parkinson’s disease.

**Table 2:** Percentage area of the GC-MS spectrum of chemical compounds found in the exhaled breath samples.

Compound	AD	PD	Control	*p*-Value
**Acetamide**	25.76 ± 3.66	20.58 ± 1.41	24.86 ± 3.78	≤0.05
**Phenol**	60.45 ± 3.87	59.88 ± 2.28	60.00 ± 6.54	ns
**Alkanes**	0.17 ± 0.13	0.21 ± 0.14	0.20 ± 0.13	ns
**Pentadecane**				
**Heptadecane**				
**Tetradecane**				
**Siloxanes**	4.58 ± 3.05	8.38 ± 2.31	4.89 ± 2.33	≤0.05
**Cyclopentasiloxane**				
**Cyclotrisiloxane**				
**1,2-benzenedicarboxylic acid diethyl ester**	0.46 ± 0.46	0.09 ± 0.04	0.32 ± 0.38	ns
**Ethyl 3-cyano-2,3-bis (2,5-dimethyl-3-thienyl)-acrylate**	0.04 ± 0.01	0	nd	≤0.05
**Triphenyl phosphate**	0.03 ± 0.03	nd	0.07 ± 0.07	≤0.05
**1-phenanthrenol**	0.04 ± 0.01	0.019 ± 0.01	nd	≤0.05

Data presented as mean ± standard deviation. The *p*-value for variables was computed using one-way analysis of variance (ANOVA). *p ≤* 0.05; ns = not significant; nd = not determined; AD, Alzheimer’s disease; PD, Parkinson’s disease.
